# Weight Loss Expectations of Adults With Binge Eating: Cross-sectional Study With a Human-Centered Design Approach

**DOI:** 10.2196/40506

**Published:** 2023-02-28

**Authors:** Claire Voss, Jianyi Liu, Angela Chang, Jacqueline A Kosmas, Abigail Biehl, Rebecca L Flynn, Kaylee P Kruzan, Jennifer E Wildes, Andrea K Graham

**Affiliations:** 1 Center for Behavioral Intervention Technologies Northwestern University Feinberg School of Medicine Chicago, IL United States; 2 Department of Psychiatry and Behavioral Neuroscience University of Chicago Chicago, IL United States

**Keywords:** binge eating, weight loss expectations, overvaluation of weight and shape, digital intervention, human-centered design, weight loss, user expectations, behavioral change, eating disorder, obesity, overweight

## Abstract

**Background:**

People tend to overestimate their expectations for weight loss relative to what is achievable in a typical evidence-based behavioral weight management program, which can impact treatment satisfaction and outcomes. We are engaged in formative research to design a digital intervention that addresses binge eating and weight management; thus, understanding expectations among this group can inform more engaging intervention designs to produce a digital intervention that can achieve greater clinical success. Studies examining weight loss expectations have primarily focused on people who have overweight or obesity. Only one study has investigated weight loss expectations among people with binge eating disorder, a population that frequently experiences elevated weight and shape concerns and often presents to treatment with the goal of losing weight.

**Objective:**

The aim of the study is to investigate differences in weight loss expectations among people with varying levels of binge eating to inform the design of a digital intervention for binge eating and weight management. Such an evaluation may be crucial for people presenting for a digital intervention, given that engagement and dropout are notable problems for digital behavior change interventions. We tested the hypotheses that (1) people who endorsed some or recurrent binge eating would expect to lose more weight than those who did not endorse binge eating and (2) people who endorsed a more severe versus a low or moderate overvaluation of weight and shape would have higher weight loss expectations.

**Methods:**

A total of 760 adults (n=504, 66% female; n=441, 58% non-Hispanic White) completed a web-based screening questionnaire. One-way ANOVAs were conducted to explore weight loss expectations for binge eating status as well as overvaluation of shape and weight.

**Results:**

Weight loss expectations significantly differed by binge eating status. Those who endorsed some and recurrent binge eating expected to lose more weight than those who endorsed no binge eating. Participants with severe overvaluation of weight or shape expected to lose the most weight compared to those with low or moderate levels of overvaluation of weight and shape.

**Conclusions:**

In the sample, people interested in a study to inform a digital intervention for binge eating and weight management overestimated their expectations for weight loss. Given that weight loss expectations can impact treatment completion and success, it may be important to assess and modify weight loss expectations among people with binge eating prior to enrolling in a digital intervention. Future work should design and test features that can modify these expectations relative to individuals’ intended treatment goals to facilitate engagement and successful outcomes in a digital intervention.

## Introduction

Human-centered design is an approach to ground interventions on the needs of the people who will be using them and the contexts in which they will be implemented [1-3]. We are engaged in a program of research applying a human-centered design approach to develop a digital intervention that addresses binge eating and weight management. Digital interventions offer an opportunity to scale effective interventions to those who need them [4], and research supports their efficacy for weight loss among people with overweight and obesity [5]. However, few studies have investigated digital interventions for weight loss among people with binge eating—a group estimated to make up between 13% and 30% of people seeking weight loss treatment [6].

People presenting for weight loss interventions tend to overestimate their expectations for weight loss. Among individuals seeking treatment to lose weight, most endorse a desire to lose more than 10% of their current body weight [7,8]. However, weight loss programs for adults typically aim to help people lose 5%-10% of their body weight [9]. Further, across trials of commercial weight loss interventions (eg, Weight Watchers, Jenny Craig, and Nutrisystem), participants lose, on average, only between 0% and 10% of their body weight [10]. When individuals are asked about their weight loss expectations, the majority report that they would be disappointed to lose between 5% and 10% of their body weight [11,12], and in one study, nearly half (47%) of the participants attained a weight loss that was less than their “disappointed” weight loss goal by the end of treatment [12].

Discrepancies between weight loss expectations and actual weight loss treatment outcomes are problematic because participants’ satisfaction with their weight loss is a determinant of weight loss maintenance, meaning people who are less satisfied with their weight loss are less successful at maintaining the weight that they lost in treatment over time [13,14]. People with obesity presenting for treatment with realistic weight loss expectations also show lower rates of dropout during treatment compared with those who had unrealistically high expectations [14]. Consequently, understanding people’s pretreatment expectations for a weight loss intervention may be important for ensuring that these individuals have greater success during and following treatment and fewer dropouts.

People who engage in binge eating have been understudied in the context of weight loss expectations in treatment. Just one study evaluated weight loss expectations among people with binge eating disorder (BED) and showed that people with BED expected to lose more weight than what expert and governmental guidelines deemed a reasonable amount of weight loss [15]. However, this study only investigated weight loss expectations among people meeting full-threshold diagnostic criteria for BED (based on the *Diagnostic and Statistical Manual of Mental Disorders, Fourth Edition* [*DSM-IV*], which had a more stringent frequency requirement for diagnosis than currently used in the *DSM-5*). It is not known what the weight loss expectations are for people with varying levels of binge eating (ie, threshold-level recurrent binge eating compared to subthreshold levels or no binge eating), who encompass a broader sample of the types of individuals who present for weight loss treatment. Understanding weight loss expectations among people who engage in binge eating more broadly is important because people with binge eating frequently experience elevated concerns about their weight and shape [16,17], have heightened eating disorder psychopathology when coupled with a desire to lose weight [18], experience excess weight gain, and commonly present for treatment for binge eating with the goal of losing weight [19], all of which can undermine weight loss treatment outcomes [20-23]. Therefore, understanding weight loss expectations among people with varying levels of binge eating is important to inform the design of interventions that can be targeted at a broader array of people presenting with binge eating.

Moreover, evaluating people’s expectations for treatment may be especially crucial for people presenting for a digital intervention. Engagement and dropout are notable problems for digital behavior change interventions [24], which means that digital interventions need designs that are engaging in order to achieve clinical impact. If people presenting to a digital intervention for weight management and binge eating have unrealistic expectations for weight loss, they may lose motivation or interest to engage in the program and be more likely to discontinue treatment early. Therefore, understanding the treatment expectations of this population can inform designs that sustain users’ motivation and engagement while appropriately shaping their expectations.

This study investigated differences in weight loss expectations among people who were interested in participating in a study that aimed to inform the design of a digital intervention for binge eating and weight management. The differences in weight loss expectations were compared among 3 groups: people who had not experienced any binge eating, people who endorsed some binge eating, and people who endorsed recurrent binge eating (diagnostic threshold level of ≥12 episodes) in the past 3 months. The association between weight loss expectations and overvaluation of weight and shape was also explored, given that overvaluation of weight and shape may be a mechanism by which people with binge eating overestimate weight loss expectations. The first hypothesis was that people who endorsed recurrent binge eating would expect to lose more weight in an intervention than people who endorsed only some or no binge eating. The second hypothesis was that people who endorsed a more severe overvaluation of weight and shape would have higher expectations for weight loss. This study is part of a larger program of formative research to design a digital intervention that addresses binge eating and weight management [25]. By understanding the needs and expectations of potential consumers of this digital intervention, such as their expectations for weight loss, a tool can be designed that most effectively meets the needs of those who will be engaging with it to ultimately ensure satisfaction and success in the digital intervention.

## Methods

### Participants and Procedure

Study procedures (recruitment, consent, and screening) were administered in dscout. Dscout is a qualitative market research platform with over 100,000 members who respond to surveys to be screened for eligibility and enrolled in research studies [26]. Members use this research platform primarily through their smartphones, which enables them to provide in-the-moment, in-context responses.

Interested individuals responded to a web-based advertisement in dscout for a study to “understand self-tracking behaviors in mobile interventions for weight management and binge eating” [27]. After providing web-based informed consent, participants completed a brief web-based screener comprising questions developed by the study team to assess eligibility, including whether these individuals would be “be willing to use an app to help you lose weight and manage your binge eating.” The screening was automatically ended if respondents endorsed that they were currently pregnant or not interested in losing weight.

As part of the web-based screen, individuals were asked to self-report their height and weight (to calculate their BMI, kg/m^2^) and report if they had ever experienced an episode of binge eating, which was defined to the respondents as “when someone eats an unusually large amount of food and feels a sense of loss of control while eating.” Those who indicated “yes” to binge eating were prompted to report how many binge eating episodes they had in the past 3 months. The respondents were also asked to report the amount of weight loss they perceived would be reasonable or achievable in 4 months, which reflects the duration of the intervention that was being designed. Finally, to assess weight and shape concerns as they related to respondents’ interest in engaging in a weight loss intervention, participants were asked: “Do any of the following statements apply to you? Check all that apply. ‘I struggle with my weight,’ ‘I have an active interest in losing weight,’ ‘I have an active interest in changing my body size,’ ‘None of the above.’” This question was used as a proxy measure of overvaluation of weight and shape given that the length of screening in dscout (ie, limited to 20 items) precluded including longer, established measures of this construct.

Of the 818 individuals who initiated the screener, 45 (5.5%) were screened out automatically (ie, did not indicate an active interest in losing weight or indicated they were pregnant). An additional 13 (1.5%) participants who indicated a current weight that would be incompatible with life (ie, ≤50 pounds [22.7 kg]) were excluded. Thus, 760 participants were included in the analyses.

### Ethics Approval

This study was approved by the Northwestern University Institutional Review Board (STU00213531). All participating individuals provided web-based informed consent. Participants were not provided compensation for completing the web-based screener.

### Analyses

Since weight loss expectations are contingent on people’s current weight, participants’ percent of expected weight loss was calculated as their expected weight loss divided by their current weight. Weight loss expectation values were imputed for 50 participants who reported a weight loss expectation that was reasonably assumed to be their goal weight rather than their expectation for weight loss (eg, an expectation to lose 190 pounds [86.2 kg] of their current weight of 200 pounds [90.7 kg]). For these individuals, their weight loss expectation was imputed as the difference between their current weight and their reported weight loss expectation value (eg, following the above example, the imputed value was 10 pounds [4.5 kg]). Analyses were run with and without these 50 participants, and results remained consistent across groups; therefore, we report on the full sample.

The BMI was calculated for each participant. The imperial equation was used: weight in pounds multiplied by 703, divided by height in inches squared. Regarding binge eating, respondents were grouped as those who endorsed no binge eating in the last 3 months, some binge eating episodes (≥1 but <12 episodes in the last 3 months), and recurrent binge eating episodes (≥12 episodes in the last 3 months). Using our proxy measure for overvaluation of weight and shape, the items participants endorsed regarding their struggles with their weight and body size were summed, yielding 3 groups: low (1 statement selected), moderate (2 statements selected), and severe (3 statements selected).

Analyses were conducted using SPSS software (version 27; IBM Corp). A 1-way ANOVA was conducted with posthoc comparisons using Tukey honestly significant difference tests to compare differences between binge eating groups on age, BMI, and weight loss expectations. A chi-square test was conducted to explore differences in the overvaluation of weight and shape severity levels by binge eating status. To explore differences in weight loss expectations by the overvaluation of weight and shape severity level, an ANOVA was conducted. A 2-way ANOVA between groups was used to explore the interaction between binge eating status and weight and shape severity levels on weight loss expectations. *P* values <.05 were considered statistically significant and η^2^ was calculated to determine effect size.

## Results

### Overview

Demographic information for the full sample (N=760) and binge eating group is presented in Table 1. Participants had a mean age of 34.4 (SD 11.3) years and a mean BMI of 29.33 (SD 7.7) kg/m^2^. There was no substantial difference between binge eating groups in age. There was a statistically significant difference in BMI between binge eating groups (*F*_2,750_=8.37, *P*<.001, η^2^=0.02), with those in the some and recurrent binge eating groups reporting significantly higher BMI scores than those with no binge eating, although this was a small effect. The majority of participants identified as female (n=504, 66.3%) and non-Hispanic White (n=441, 58%).

**Table 1:** Demographic information for the full sample and by binge eating group.

Characteristic		Full sample (N=760)	Recurrent binge eating (n=199)	Some binge eating (n=318)	No binge eating (n=243)
Age (years), mean (SD)		34.4 (11.3)	34.3 (11.1)	35.0 (11.3)	33.9 (11.5)
BMI (kg/m^2^), mean (SD)		29.3 (7.7)	30.1 (7.2)	30.1 (8.5)	27.7 (6.7)
**Gender, n (%)**					
	Female	504 (66.3)	121 (60.8)	222 (69.8)	161 (66.2)
	Male	242 (31.8)	75 (37.7)	89 (28.0)	78 (32.1)
	Nonbinary	7 (0.9)	1 (0.5)	3 (0.9)	3 (1.2)
	Transgender man	2 (0.2)	0 (0.0)	1 (0.3)	1 (0.4)
	Prefer not to say	5 (0.7)	2 (1.0)	3 (0.9)	0 (0.0)
**Race or ethnicity, n (%)**					
	White	441 (58.0)	109 (54.8)	196 (61.6)	136 (56.0)
	Black	126 (16.6)	35 (17.6)	48 (15.1)	43 (17.7)
	Hispanic	81 (10.6)	23 (11.6)	31 (9.7)	27 (11.1)
	Asian	71 (9.3)	20 (10.1)	27 (8.5)	24 (9.9)
	American Indian or Alaskan Native	5 (0.7)	0 (0.0)	4 (1.3)	1 (0.4)
	Middle Eastern or North African	10 (1.3)	4 (2.0)	1 (0.3)	5 (2.1)
	Pacific Islander or Native Hawaiian	1 (0.1)	1 (0.5)	0 (0.0)	0 (0.0)
	Others	1 (0.1)	0 (0.0)	0 (0.0)	1 (0.4)
	Prefer not to say	12 (1.6)	3 (1.5)	4 (1.3)	5 (2.1)
	Did not answer	12 (1.6)	4 (2.0)	7 (2.2)	1 (0.4)
Percentage weight loss expectations, mean (SD)		10.4 (5.9)	11.1 (4.8)	10.8 (6.1)	9.4 (6.2)
**Overvaluation of weight and shape, n (%)**					
	Low	187 (24.6)	25 (12.6)	67 (21.1)	95 (39.1)
	Moderate	166 (21.8)	31 (15.6)	65 (20.4)	70 (28.8)
	Severe	407 (53.6)	143 (71.9)	186 (58.5)	78 (32.1)

### Weight Loss Expectations

There was a statistically significant difference in weight loss expectations by binge eating status, although the effect size was small (*F*_2,757_=6.08, *P*=.002, η^2^=0.016). Posthoc comparisons indicated that participants who endorsed recurrent binge eating expected to lose the most weight (11.1% ±4.8% of their weight in 4 months), followed by participants who endorsed some binge eating (10.8% ±6.1% of their weight), and participants who endorsed no binge eating (9.4% ±6.2% of their weight). The mean score for those with no binge eating was significantly different from those with some binge eating (*P*=.01) and those with recurrent binge eating (*P*=.006). There was no statistically significant difference in weight loss expectations between those who endorsed some binge eating and those who endorsed recurrent binge eating. Controlling for BMI, the difference in weight loss expectations by binge eating status was slightly attenuated with trend level significance and small effect size (*F*_2,749_=2.82, *P*=.06, η^2^=0.01).

### Relationship Between Overvaluation of Weight and Shape, Binge Eating, and Weight Loss Expectations

There was a statistically significant association between binge eating status and overvaluation of weight and shape, with a medium effect size (χ^2^_4_ [n=760]=78.12, *P*<.001, Φ=0.32), indicating more binge eating was associated with greater severity of overvaluation of weight and shape, as shown in Table 1.

There also was a statistically significant difference in weight loss expectations by the overvaluation of weight and shape severity, with a medium-to-large effect size (*F*_2,757_=42.87, *P*<.001, η^2^=0.11). Posthoc comparisons showed that participants who endorsed severe overvaluation of weight and shape expected to lose more weight (12.1% ±5.5% of their body weight in 4 months) than participants who endorsed moderate overvaluation of weight and shape (9.4% ±6.3% of their weight) and low overvaluation of weight and shape (7.7% ±5.0% of their weight). The mean score for participants with severe overvaluation of weight and shape was significantly different from those with moderate overvaluation of weight and shape (*P*<.001) and those with low overvaluation of weight and shape (*P*<.001). Similarly, the mean score for participants who endorsed moderate overvaluation of weight and shape differed significantly from participants who endorsed low overvaluation of weight and shape (*P*=.02). Controlling for BMI, the difference in weight loss expectation by the overvaluation of weight and shape severity remained significant, with a medium effect size (*F*_2,749_=23.25, *P*<.001, η_p_^2^=0.06).

The interaction effect between binge eating status and overvaluation of weight shape on weight loss expectations was not statistically significant, using the more stringent *P* value of <.01, given that the Levene test of equality of error variances was significant (*F*_2,751_=2.47, *P*=.04, η_p_^2^=0.013). There was a statistically significant main effect for overvaluation of shape and weight, with a medium-to-large effect size (*F*_2,751_=31.075, *P*<.001, η_p_^2^=0.076). The main effect for binge eating status did not reach statistical significance (*F*_2,751_=0.695, *P*=.50, η_p_^2^=0.002).

## Discussion

### Principal Findings

Given that people presenting for weight loss treatment commonly overestimate their expectations for weight loss [7], it is important to understand whether the presence of binge eating contributes to or exacerbates this problem, which could have implications for the design of digital interventions for people with binge eating. This study examined whether people who endorse binge eating, compared to people without binge eating, expect to lose more weight in interventions for weight management and binge eating, and whether more severe overvaluation of weight and shape is associated with higher weight loss expectations. Findings confirmed our hypotheses, as we found that higher weight loss expectations were associated with both increased overvaluation of weight and shape and more frequent binge eating. In addition, the substantial main effect of overvaluation of weight and shape in a test of the interaction between these constructs suggests that overvaluation of weight and shape contributes to weight loss expectations even beyond the presence of binge eating.

To date, only one study has investigated weight loss expectations among people with binge eating, and it showed that people with BED are expected to lose more weight than is recommended by experts and governmental guidelines [15]. This study extended the prior work by documenting that individuals who endorsed any binge eating (ie, some binge eating and recurrent binge eating) expected to lose more weight in treatment than people who did not endorse binge eating. In general, people presenting for weight loss treatment often expect to lose more weight than is typically achievable [7], and this study showed that the presence of binge eating may exacerbate this expectation. We also found that these individuals, on average, expected to lose more than 10% of their body weight, which exceeds what is typically achieved in behavioral weight loss programs [9,10]. These findings highlight the importance of shaping the expectations for weight change that individuals with binge eating can likely achieve in treatment while sustaining their interest in pursuing behavior change.

Indeed, modifying these expectations may contribute to better overall engagement with a digital intervention. Digital interventions face engagement challenges [24,28], in part because they lack some of the cues and features that face-to-face treatment with a practitioner offers. This means that digital interventions have greater pressure to use designs that “get it right” in meeting consumers’ needs, preferences, and goals. Because unrealistic weight loss expectations have been associated with lower treatment satisfaction and outcomes in face-to-face weight loss programs [13,14], a digital intervention for weight management must be designed effectively to support this misalignment. This could include designs that help people with binge eating adjust their expectations before or at the start of initiating the digital intervention, as well as features within the intervention that consistently promote weight change expectations that are realistic and achievable, such as in the app’s content and self-monitoring tools.

As a more detailed example of a potential feature, consider that behavioral interventions often index people’s goals at the beginning of treatment as a way to increase motivation and sustain engagement. Knowing that people with binge eating are likely to select goals that are not aligned with established recommendations for weight loss, one way to reduce later discrepancies is for the intervention to provide users with goal prompts that model realistic weight change and other health outcomes as well as psychoeducation on the intent of the intervention in building sustainable changes to promote long-term health [29]. This feature could facilitate user agency while also ensuring that a user’s goals are aligned with what is typical in a weight loss program for this population. Similarly, psychoeducation around why these prompts were provided may help users understand the clinical rationale behind these goals. Future work will need to investigate these intervention design ideas, as well as others that are effective for modifying these expectations and acceptable to people with binge eating.

### Limitations

The strengths of this study include the large sample of people with varying levels of binge eating recruited from across the United States. Though these findings were exploratory as a secondary analysis, they extend our understanding of the impact of binge eating on weight loss expectations, which is important because unrealistic expectations could have implications for individuals’ success in treatment. Study limitations should also be noted. Because this was a secondary analysis of screening data for enrollment into a subsequent study, our screening measure was self-created for the purpose of screening and therefore was not a validated tool, which included a proxy measure of overvaluation of weight and shape. Assessment of binge eating was via self-report, and self-reported binge eating can differ from objective measures [30] as individuals may not be able to correctly assess a binge eating episode relative to the clinical definition or can misjudge the frequency of binge eating behaviors. Self-reported height and weight, as was assessed in our screener, can also differ from objective measurements, although there is generally strong agreement between self-reported weights and weights measured using clinic or electronic scales that transfer data back to researchers [31-33]. Additionally, we did not assess for BED or other psychiatric diagnoses since the presence and frequency of binge eating (not BED) was the focus of the study for which respondents were completing the screener. The study was conducted on the internet, and there have been recent concerns with the validity of participants’ responses in web-based research platforms for eating disorder research [34], although this has not been shown for the platform we used. Lastly, participants were recruited for a study to understand self-tracking behaviors in mobile interventions for binge eating and weight management, but they were not presenting for treatment. Therefore, it may be beneficial to assess weight loss expectations among people with binge eating who are enrolling in digital interventions for binge eating and weight management.

### Conclusions

In this study, we showed that weight loss expectations were higher among people with binge eating (some or recurrent) compared to those with no binge eating, and those with severe overvaluation of weight and shape experienced higher weight loss expectations than those with low to moderate levels of overvaluation. As a next step in the design process to create a digital intervention for managing binge eating and weight, findings indicate that future work should now focus on designing and testing strategies and features that can modify weight loss expectations relative to individuals’ intended treatment goals while still maintaining their motivation to change their behavior. Indeed, addressing weight change expectancies explicitly and early on may increase the likelihood that an individual will engage with a digital intervention fully and positively impact treatment outcomes.

## Abbreviations

BED: binge eating disorder

## Data Availability

The data analyzed for this study are available from the corresponding author on reasonable request.
